# P09 Broadening horizons: the use of OPAT to facilitate outpatient faecal transplants

**DOI:** 10.1093/jacamr/dlaf239.013

**Published:** 2026-01-05

**Authors:** Thomas Harrison, Clare Shaw, Beatrix Teo, Laura Prtak, Katharine Cartwright

**Affiliations:** Sheffield Teaching Hospital, Sheffield, UK; Sheffield Teaching Hospital, Sheffield, UK; Sheffield Teaching Hospital, Sheffield, UK; Sheffield Teaching Hospital, Sheffield, UK; Sheffield Teaching Hospital, Sheffield, UK

## Abstract

**Background:**

Faecal microbiota transplantation (FMT) is a highly effective treatment for *Clostridioides difficile* infection and is typically administered via a nasogastric tube requiring inpatient admission. In line with forthcoming national OPAT guidelines promoting diversification of services beyond antimicrobial delivery, an OPAT protocol was developed and implemented to support safe and effective outpatient administration of FMT.

**Methods:**

Baseline review of CDI cases in the year 2022–23 identified 15 of 37 (40%) patients meeting FMT inclusion criteria; only four had a documented discussion and one received treatment. Among patients treated with FMT over the past decade, 10 of 12 (83%) met OPAT eligibility. Process mapping highlighted unnecessary inpatient stays, over-dependence on a single coordinator, and the high burden of clerking and post-take documentation. A multidisciplinary team subsequently developed and implemented a pilot protocol for OPAT FMT delivery based on national guidance.

**Results:**

During the first year of implementation, four FMT procedures were performed. All patients met national eligibility criteria and underwent treatment within 18 days of referral, with no recurrences of *C. difficile* infection. Patient feedback questionnaire was very positive. Formal staff feedback after the first two procedures identified areas for improvement, including assigning a dedicated nurse to coordinate the FMT, having the referring clinician complete request forms for FMT material, reserving a doctor’s slot for consent, and clarifying reception booking processes. These modifications were applied to subsequent procedures.

**Challenges and lessons learned:**

Implementation of outpatient FMT has been constrained by several operational and financial factors. The cost of the procedure exceeds £1000, while reimbursement for outpatient delivery is lower than the procedure cost, in contrast to inpatient admission where reimbursement is higher. As a result, the department currently operates at a financial deficit, limiting the number of procedures that can be offered annually. The limited availability of side rooms necessitates cancellation of PICC insertion slots to accommodate FMT procedures, reducing overall throughput. Expanding the service to accept referrals from general practitioners and other hospitals that do not currently perform FMT could increase patient access while supporting continued development of staff expertise. Future updates to national FMT protocols, including the potential use of oral FMT capsules, may eliminate the need for nasogastric administration and further facilitate outpatient delivery.

**Conclusions:**

Outpatient FMT via an OPAT pathway is feasible, safe, and well-received, aligning with the national drive for service diversification and demonstrating positive patient outcomes.

